# Editorial: Innovative approaches to catalyze preclinical and clinical research on amyotrophic lateral sclerosis (ALS) and related disorders

**DOI:** 10.3389/fnins.2025.1582539

**Published:** 2025-03-18

**Authors:** Thibaut Burg, Laura Tzeplaeff, Raphaelle Cassel, Paul Lingor

**Affiliations:** ^1^Department of Neurosciences, Experimental Neurology and Leuven Brain Institute (LBI), KU Leuven-University of Leuven, Leuven, Belgium; ^2^Laboratory of Neurobiology, Center for Brain & Disease Research, VIB, Leuven, Belgium; ^3^Clinical Department of Neurology, School of Medicine, rechts der Isar Hospital, Technical University of Munich, Munich, Germany; ^4^UMR-S 1329, Strasbourg Translational Neuroscience & Psychiatry STEP-CRBS, University of Strasbourg, INSERM, Strasbourg, France; ^5^German Center for Neurodegenerative Diseases (DZNE), Munich, Germany; ^6^Munich Cluster for Systems Neurology (SyNergy), Munich, Germany

**Keywords:** Amyotrophic lateral sclerosis (ALS), motor neuron, neurodegeneration, clinical research, biomarker, therapy

## Introduction

Amyotrophic lateral sclerosis (ALS) stands as a rare disease associated with a substantial socioeconomic burden and a lifetime risk of around 1/350 (Heinrich et al., 2023; Ryan et al., 2019). Characterized by the degeneration of motor neurons (MNs), ensuing muscle weakness, and progressive paralysis, ALS typically results in a devastatingly short survival time of 2–5 years after the initial diagnosis (Masrori and Van Damme, 2020). A major challenge in ALS research stems from the disease's remarkable heterogeneity. This heterogeneity manifests in various aspects, including clinical presentation, rate of progression, genetics, and underlying pathophysiological mechanisms, making it difficult to develop universally effective treatments (Goyal et al., 2020). The urgent need for accelerated research is evident, as enhancing our understanding of ALS holds the promise of unraveling broader insights into neurodegenerative processes. This Research Topic brings together studies that push the boundaries of our understanding and pave the way for novel therapeutic strategies.

## Beyond motor neuron degeneration

ALS is increasingly recognized as a multisystem disease, extending beyond its traditional classification as an MN disorder. Recent research has revealed that ALS affects various non-motor systems, including cognitive, behavioral, autonomic, and sensory functions (Masrori and Van Damme, 2020). Shi et al. employed bidirectional two-sample Mendelian randomization (MR) using genome-wide association study data for ALS and various brain structures to establish causal relationships between brain structural changes and ALS risk. They show that morphometric changes, such as cortical surface area or cortical thickness, are associated with the risk for ALS. In addition, extramotor atrophy in the temporal lobes further supports the mechanistic similarities of ALS with frontotemporal dementia.

Knowledge of the cell types involved in ALS pathogenesis is an essential step to understanding the complete picture of the disease, as non-cell autonomous mechanisms contribute to MN vulnerability (Schweingruber and Hedlund, 2022). Goffin et al. shed light on an often-overlooked cell population: spinal interneurons. Their comprehensive review highlights how these cells, which regulate MN activity, may contribute to disease onset and progression. The authors proposed the working hypothesis that functional interactions between spinal interneurons and MNs are dysregulated before MN degeneration or symptom onset. These changes may stem from intrinsic defects or compensatory mechanisms to subtle MN disturbances. This hypothesis underscores the intricate nature of ALS pathogenesis, involving multiple cellular components and mechanisms beyond MNs alone.

## Biomarkers of disease progression

Identifying and characterizing biomarkers of disease progression is crucial for ALS, as they help stratify patients into homogeneous groups, allowing for more effective clinical trials with sensitive detection of therapeutic effects. Prognostic biomarkers aid in tailoring treatment to individual progression rates, permitting more accurate and personalized care, ultimately leading to better outcomes for ALS patients (Witzel et al., 2022).

Hong et al. provide additional evidence for the role of systemic inflammation in ALS progression. Their study on Chinese ALS patients reveals that markers such as neutrophil-to-lymphocyte ratio (NLR), platelet-to-lymphocyte ratio (PLR), lymphocyte-to-monocyte ratio (LMR), and systemic immune-inflammation index (SII) serve as independent predictors of rapid disease progression. The researchers found that NLR, PLR, and SII were significantly higher in ALS patients compared to controls, while LMR was lower. Importantly, higher NLR and lower LMR were associated with shorter survival time. These inflammation markers are highly dependent on race, sex, and age (Walsh et al., 2023). Hence, further multi-center studies with larger sample sizes and more varied populations are required to validate their clinical potential.

The D50 model describes the disease course of individual patients as a sigmoidal curve from full health to complete functional loss (Steinbach et al., 2020). Meyer et al. compared three different measures of disease progression speed: D50 (overall disease aggressiveness), cFL (calculated functional loss-rate), and DPR (disease progression-rate) in ALS patients. The authors demonstrated the advantage of the D50 model in quantifying disease aggressiveness and its robust correlation with cerebrospinal fluid (CSF) levels of phosphorylated Neurofilament-Heavy-chain (pNfH) compared to other methods. Interestingly, CSF pNfH concentration was independent of the disease phase but strongly correlated with overall disease aggressiveness as quantified by D50. Although independent multi-center studies are necessary to replicate the results, these findings reinforce the potential of CSF pNfH levels as a robust prognostic marker.

## Novel therapeutic approaches

Finally, Berthiaume et al. present promising preclinical results for ATH-1105, a small-molecule positive modulator of the hepatocyte growth factor (HGF) signaling system. The authors assessed the effects of the drug in rat primary neurons *in vitro* and in a transgenic rodent model (hemizygous Prp-TDP43^A315T^ mice) *in vivo*. ATH-1105 not only promoted cell survival pathways, manifested by phosphorylation of MET, Akt, and Erk, but also attenuated Glutamate- and LPS-induced toxicity. Motor function as well as histological readouts, were improved in the animal model, supporting further analyses of ATH-1105 as a promising therapeutic for ALS.

## Conclusion

The diverse approaches presented in this Research Topic reflect the multifaceted nature of ALS research. This Research Topic impressively highlights the current focal points in the field of ALS research: studies on the understanding of disease mechanisms, biomarker investigations, and new therapeutic approaches. We hope that this Research Topic will further stimulate engagement with this important field and draw more scientific attention to this rare but severe disease.

## References

[B1] Goyal N. A. Berry J. D. Windebank A. Staff N. P. Maragakis N. J. van den Berg L. H.. (2020). Addressing heterogeneity in amyotrophic lateral sclerosis CLINICAL TRIALS. Muscle Nerve. 62, 156–166. 10.1002/mus.26801 31899540 PMC7496557

[B2] Heinrich F. Cordts I. Günther R. Stolte B. Zeller D. Schröter C.. (2023). Economic evaluation of Motor Neuron Diseases: a nationwide cross-sectional analysis in Germany. J. Neurol. 270, 4922–4938. 10.1007/s00415-023-11811-1 37356024 PMC10511618

[B3] Masrori P. Van Damme P. (2020). Amyotrophic lateral sclerosis: a clinical review. Eur. J. Neurol. 27, 1918–1929. 10.1111/ene.14393 32526057 PMC7540334

[B4] Ryan M. Heverin M. McLaughlin R. L. Hardiman O. (2019). Lifetime risk and heritability of amyotrophic lateral sclerosis. JAMA Neurol. 76, 1367–1374. 10.1001/jamaneurol.2019.2044 31329211 PMC6646974

[B5] Schweingruber C. Hedlund E. (2022). The cell autonomous and non-cell autonomous aspects of neuronal vulnerability and resilience in amyotrophic lateral sclerosis. Biology 11:1191. 10.3390/biology11081191 36009818 PMC9405388

[B6] Steinbach R. Batyrbekova M. Gaur N. Voss A. Stubendorff B. Mayer T. E.. (2020). Applying the D50 disease progression model to gray and white matter pathology in amyotrophic lateral sclerosis. Neuroimage Clin. 25:102094. 10.1016/j.nicl.2019.102094 31896467 PMC6940701

[B7] Walsh C. P. Lindsay E. K. Grosse P. Natale B. N. Fairlie S. Bwint A.. (2023). systematic review and meta-analysis of the stability of peripheral immune markers in healthy adults. Brain Behav. Immun. 107, 32–46. 10.1016/j.bbi.2022.09.011 36152782 PMC9729419

[B8] Witzel S. Mayer K. Oeckl P. (2022). Biomarkers for amyotrophic lateral sclerosis. Curr. Opin. Neurol. 35, 699–704. 10.1097/WCO.0000000000001094 35942674

